# The Landscape and Prognosis Potential of the T-Cell Repertoire in Membranous Nephropathy

**DOI:** 10.3389/fimmu.2020.00387

**Published:** 2020-03-10

**Authors:** Yu Zhang, Yabin Jin, Zhanwen Guan, Huishi Li, Zuhui Su, Chao Xie, Xiangping Chen, Xiaofen Liu, Yingming Pan, Peiyi Ye, Lifang Zhang, Yaozhong Kong, Wei Luo

**Affiliations:** ^1^Nephrology Department, The First People's Hospital of Foshan, Foshan, China; ^2^Clinical Research Institute, The First People's Hospital of Foshan, Foshan, China

**Keywords:** T-cell receptor repertoire, membranous nephropathy, clinical response, high-throughput sequencing, non-invasive biomarkers

## Abstract

Membranous nephropathy (MN), a common pathological type of adult nephrotic syndrome, is an antibody-mediated kidney disease. It is widely accepted now that MN is an immune-related disease that involves the whole immune system. In this study, we analyzed the T-cell receptor beta chain (TCRβ) repertoire of the circulating T lymphocytes of MN patients and healthy controls using high-throughput sequencing. We compared multiple aspects of the TCRβ repertoire, including diversity and the Vβ and Jβ genes between MN patients and healthy controls, and we found that the diversities within the VJ cassette combination in the peripheral blood of MN patients were lower than in the healthy controls. We also found the TCRβ repertoire similarity between pre- and post-therapy could reflect the clinical outcome, and two Vβ genes in pre-therapy had the potential to predict the therapeutic effect. These findings indicated the potential of the TCRβ repertoire as non-invasive biomarkers for the prognosis prediction of MN. The characteristics of circulating T-lymphocyte repertoires shed light on MN detection, treatment, and surveillance.

## Introduction

Membranous nephropathy (MN), a common pathological type of adult nephrotic syndrome, is an antibody-mediated kidney disease clinically characterized by proteinuria (1). The typical pathological characteristics are the deposition of immune complexes on the subepithelial side of the glomerular basement membrane (GBM), resulting in diffuse thickening of GBM (2, 3). Recently, a large multicenter retrospective study, which investigated 71,151 cases of renal puncture in China from 2004 to 2014, found that the proportion of MN was 23.4%, only lower than IgA nephropathy, and was growing rapidly, and this was associated with long-term exposure to air pollution (4).

MN was regarded as an organ-specific autoimmune disease in which IgG autoantibodies form subepithelial immune complexes with autoantigens expressed on podocyte cell surfaces (2, 3). The autoantibodies targeting phospholipase A2 receptor (PLA2R) and thrombospondin type-1 domain-containing 7A (THSD7A) were found in some MN patients (5, 6). It is thus widely accepted that MN is an immune-related disease with the involvement of the whole immune system.

The diagnosis of MN relied on the histopathology of renal biopsy, which was examined by light microscopy, immunofluorescence, and electron microscopy (7). It was invasive and could lead to infection and potential damage to the kidney. Performing it repeatedly during and after treatment to monitor the disease progress was consequently not practicable. In recent years, many novel biomarkers have been developed in plasma or urine. Of all them, the most commonly used was APLA2R (8). Although APLA2R showed some potential, there are still considerable false positives and false negatives.

At present, studies of MN have focused on the B-cell immunity, and rituximab is currently the most widely used anti-CD20 monoclonal antibody. Some studies also found that there was a correlation between MN and T cells. Previous studies have shown that there has been an increase in the CD4/CD8 subset ratio of some patients with MN with or without nephrotic syndrome (9–11), and a recent study reported that the evaluation of Tregs in patients with severe idiopathic MN could predict an early response to rituximab (12). These studied indicated that the pathogenesis of MN was also closely related to T cells.

Recently, using T-cell receptor repertoire high-throughput sequencing (TCR-HTS), a series of studies, including our own, have characterized the signatures of T-cell repertoires and revealed their diagnostic and prognostic implications in various types of immune-mediated diseases, including infectious diseases (13, 14), autoimmune diseases (15–17), and tumors (18–20). The TCR repertoire could reflect the status of T-cell immunity in MN, and investigating the diversity of T lymphocytes by TCR-HTS could help understand the pathogenesis of MN.

In this study, we analyzed the T-cell receptor beta chain (TCRβ) repertoire of the circulating T lymphocytes of MN patients (pre-therapy and post-therapy) and healthy controls using TCR-HTS. We compared multiple aspects of the TCRβ repertoire, including diversity and Vβ and Jβ genes between MN patients and healthy controls, and we found that the diversities of VJ cassette combination in peripheral blood of MN patients were lower than healthy controls. We also found the TCRβ repertoire similarity between pre- and post-therapy could reflect the clinical outcome and two Vβ genes in pre-therapy also had the potential to predict the therapeutic effect.

## Materials and Methods

### Sample Collection

This study was approval by the ethics committee of the Affiliated Foshan Hospital of Sun Yat-Sen University. Twenty patients with membranous nephropathy were recruited in this study from 2016 to 2018. Clinical characteristics of the patients are showed in Table 1 and Table S1. The inclusion criteria were MN diagnosis established by renal biopsy. The renal biopsy specimens of all the patients were examined by light microscopy, immunofluorescence, and electron microscopy. Most of the patients accepted the therapy with prednisone (1 mg/kg body weight) or tacrolimus (0.05 mg/kg body weight, two patients with contraindication to prednisone) once per day for about 6 months. But one of the patients rejected the treatment because of insurance issues, and they were thus excluded. Peripheral blood samples were collected before therapy initiation and after about 6 months of treatment for all the patients. The laboratory assessment included APLA2R status, serum albumin, proteinuria, 24-h urine protein (24 hUP), urea nitrogen, creatinine, and eGFR levels, and these were collected at the time of renal biopsy and at 6-months follow-up. We evaluated the therapeutic effects of the patients according to KIDNEY DISEASE IMPROVING GLOBAL OUTCOMES (KDIGO) (21, 22). We also recruited 19 healthy volunteers without a history of cancer, autoimmune disorder, or surgery as a control, and blood samples were drawn from each healthy volunteer. Peripheral blood mononuclear cells (PBMCs) were isolated from fresh anticoagulant peripheral blood by density gradient centrifugation, lysed with TRIzol® reagent (Life, US), and frozen at −80°C until further processing.

**Table 1:** Summary of clinical and laboratory information of the recruited people.

**Variable**		**MN patients**	**Healthy control**	***P*-value**
Gender (Female/male)		7/12	7/12	1.0
Age (years)	Median (range)	48 (16-67)	47 (23-68)	0.93
24 h UP (g)		5.1 (1.7–15.9)	/	/
Serum creatinine (μmol/l)		75 (49–123)	/	/
Serum urea nitrogen (mmol/l)		5.5 (3.4–9.7)	/	/
eGFR (ml/min/1.73 m^2^)		73.8 (52.2–108.5)	/	/
APLA2R (Ru/ml)		109.7 (1.19–1500.00)	/	/
Serum albumin(g/l)		24.7 (15.7–35.3)	/	/

### High-Throughput Sequencing of TCRβ Chains

Total RNA was extracted from PBMC lysates using a total RNA Kit (OMEGA Bio-tek, US) according to the manufacturer's instructions. For every sample, 1 μg of total RNA was reverse transcribed into 5′ RACE-ready cDNA using a SMARTer PCR cDNA synthesis kit (Clontech, US). In order to amplify the completely arranged TCRβ fragments, the first-strand cDNA was used as templates for a 5′RACE-PCR with forward universal primer and reverse primer specific to the TCRβ constant (C) region (5′-AACACSTTKTTCAGGTCCT-3′). The PCR reaction conditions were the same as in our previous paper: 94°C for 3 min, carried out with 35 cycles of denaturing at 94°C for 15 s, annealing at 58°C for 30 s, extension at 72°C for 45 s, and a final extension at 72°C for 10 min. The PCR products were purified by 2% agarose gel electrophoresis and the gel extraction kit (QIAGEN, German). The Illumina Hiseq sequence adaptors were ligated to construct sequencing libraries, which were then sequenced on an Illumina Hiseq2500 platform. The raw data of the sequencing will be available on request.

### Bioinformatics Analysis of TCRβ Repertoire Data

The sequencing data were stored in a FASTQ format. Firstly, the low-quality sequences were filtered according to four strict criteria: (1) contaminated by the adapter sequence; (2) had more than 5% uncalled bases (N); (3) had an average Phred-type Q-score <15; and (4) had PE reads with low-quality base readings(Q-score <10) at the ends of reads. Secondly, the high-quality sequences were aligned with the TCRβ reference genes by BLAT (-stepSize=5 –minIdentity = 0 –minScore = 0) (23). The TCR reference gene sequences were downloaded from the IMGT/GENE database (24). If V, J, and C genes in a given sequence were all identified, we further translated them into an amino acid (aa) sequence. The aa sequences without a terminator were selected as the productive TCR sequences. Lastly, at the V–D–J junctions, the sequences that started with cysteine and ended with the FGXG motif [C…FGXG] were defined as CDR3 (23).

### Diversity Indices

We selected the Shannon entropy as our fundamental measure of the diversity; it has been widely used in previous TCR repertoire studies for its inclusion of both richness (number of members) and diversity (evenness of distribution) and its applicability to more complex models of repertoire size (25–27). The formula of the Shannon entropy index is as follows

$$\begin{array}{r} \text{Shannon entropy}=-\sum_{i}\left(\frac{n_{i}}{N}\right)\log_{2}\left(\frac{n_{i}}{N}\right) \end{array}$$

where i is an index that is chosen between 1 and the number of species s, n_i_ is the number of sequencing reads in species i, and N is the total number of reads.

### Similarity Between Two TCRβ Repertoires

To evaluate changes in the TCR repertoire after therapy, we calculated the Morisita-Horn similarity index (MH) of two different TCR repertoires. The index between two TCR repertoires was calculated based on the number of shared sequences between the two samples and the contribution of the shared sequences to each repertoire, and the index ranged from 0 to 1 (28, 29). The formula is as follows

$$\begin{array}{r} MH=\frac{2\sum_{i=1}^{s}x_{i}y_{i}}{\left(\frac{\sum_{i=1}^{s}x_{i}^{2}}{X^{2}}+\frac{\sum_{i=1}^{s}y_{i}^{2}}{Y^{2}}\right)XY} \end{array}$$

where *x*_*i*_ is the number of times clonotype *i* is represented in the total *X* sequences from one sample, *y*_*i*_ is the number of times clonotype *i* is represented in the total *Y* sequences from another sample, and *S* is the number of unique clonotypes.

### Statistical Analysis

GraphPad Prism version 5.1 (GraphPad Software, Inc., SanDiego, CA, USA) and SPSS 20.0 (SPSS, Inc., Chicago, IL, USA) were used for statistical analysis of the data. Comparisons between groups were conducted using the Mann–Whitney *U* test or the Wilcoxon signed ranks test if appropriate, and *P* < 0.05 were considered statistically significant. The receiver operating characteristic (ROC) curve was used to illustrate the diagnostic ability of a binary classifier system. The area under the curve (AUC) was calculated by the Hanley and McNeil method.

## Results

### Clinical and Pathological Characteristics of the Patients

The demographic and clinical characteristics data of the MN patients is summarized in Table 1. For MN patients, 12 of them were male and seven were female. The average age of the patients was 48 years old. There were no significant differences in age (*P* = 0.93) or gender (*P* = 1.0) between the MN patients and healthy controls (Figure S1). The median 24-h proteinuria was 5.1 g for patients with MN and varied largely among patients (1.7 g/24 h−15.9 g/24 h). The serum creatinine, serum urea nitrogen, serum albumin, eGFR, and APLA2R also varied extensively among MN patients, with a median of 75 μmol/l, 5.5 mmol/l, 24.7 g/l, 73.8 ml/min/1.73m^2^, and 109.7 Ru/ml, respectively.

### Profiling of the TCRβ Sequencing Data

A total of 83,414,134 productive amino acid sequences were obtained from 57 blood samples of the patients and healthy control with an average of 1,463,406 productive sequences generated per sample. The average number of productive unique sequences per sample was 96,934. All the 65 distinct Vβ and 13 distinct Jβ segments were identified, and the usage frequencies of these segments were analyzed in each sample, which are listed in Table S2. The five most frequent genes detected in almost all the samples were Vβ5.1, Vβ20.1, Vβ12.4, Vβ7.9, and Vβ29.1 as well as Jβ1.1, Jβ2.1, Jβ2.7, Jβ1.2, and Jβ2.3.

### Lower Diversity of VJ Cassette Combination in Peripheral Blood of MN Patients

**Figure 1:**
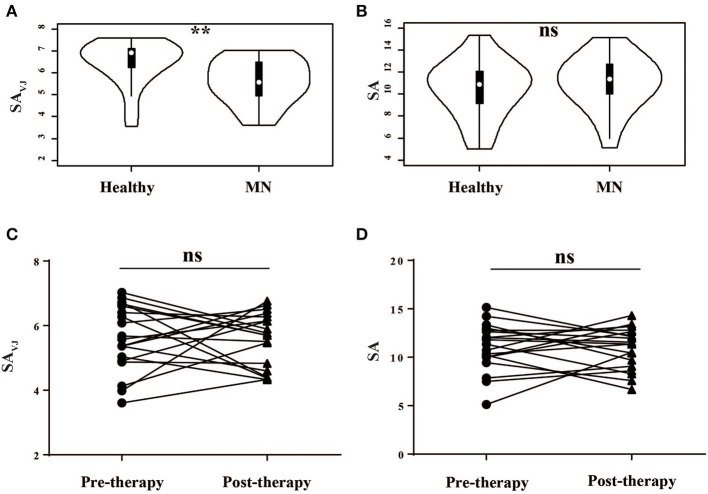
The Shannon's entropy of peripheral blood TCR repertoire between different groups. The Shannon's entropy of clonotype (SA) and VJ combination (SA_VJ_) between healthy controls and MN patients **(A,B)** and between pre- and post-therapy **(C,D)**. **P < 0.01. ns, no significant.

As a measure of diversity, we calculated the Shannon entropy—a function of the number of unique elements in the population and their frequencies—for VJ cassette combinations (SA_VJ_) and clonotypes (SA) (27). We firstly compared the diversity between MN patients and healthy controls, and the VJ combination diversity in MN patients was significantly lower than that in healthy controls (*P* = 0.003, Figure 1A), which indicated antigenic stimulation and expansion of some specific VJ combinations. No significant difference was, however, observed by clonotype diversity (*P* = 0.448, Figure 1B), which might be due to the increased random insertions and deletions independent of VJ combinations in MN patients.

We then analyzed the correlation between the diversity and clinical characteristics (Figure S2), and we compared the diversity between pre-therapy and post- therapy (Figures 1C,D)—no significant difference was observed. Moreover, we did not observe the relationship between diversity change and clinical response.

### The Different Usage Patterns of Vβ, Jβ Genes and CDR3 Motifs in MN Patients

**Figure 2:**
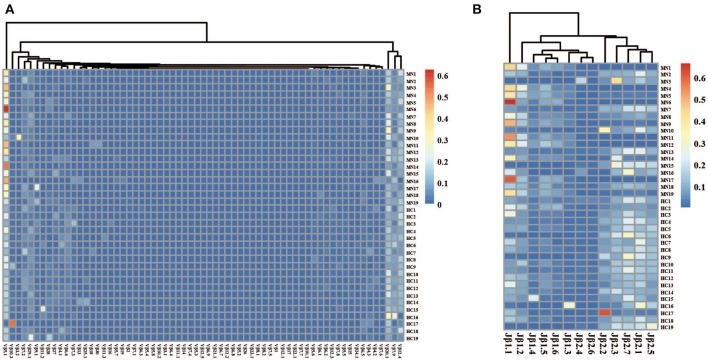
The heat maps of Vβ **(A)** and Jβ **(B)** gene usage frequencies in peripheral blood TCR repertoire of each sample from the MN patients and healthy controls.

**Figure 3:**
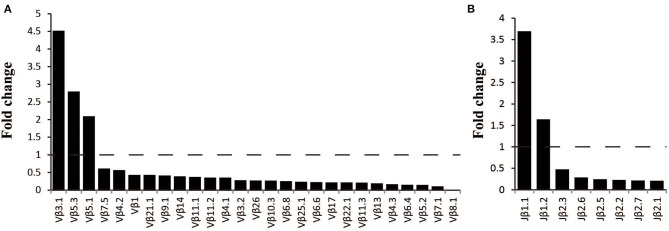
Comparison of Vβ and Jβ gene segments used in MN patients and healthy controls. **(A)** A total of 27 Vβ gene segments and **(B)** 8 Jβ gene segments with different usage frequencies between MN patients and healthy controls (all *P* < 0.05). Fold change=the median frequency in MN patients divided by the median frequency healthy controls.

**Figure 4:**
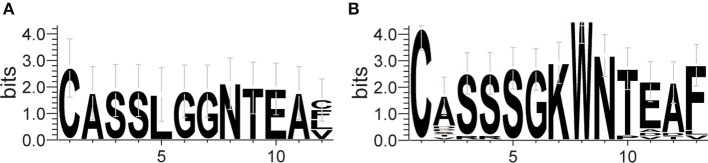
Sequence logo figures of the CDR3 motifs associated with MN. Each logo consists of stacks of symbols, with one stack for each amino acid in the sequence. The overall height of the stack indicates the degree of sequence conservation at that position, while the height of the symbols within the stack indicates the relative frequency of each amino acid at that position. The width of the stack is proportional to the fraction of valid symbols at that position.

The frequency of the TCR Vβ and Jβ genes in MN patients and healthy controls were delineated in heat maps (Figures 2A,B). Consistent with previous reports of other researchers (30–32), some Vβ genes exhibited high-frequency usage in almost all of the samples, such as Vβ5.1 and Vβ20.1. Comparing the Vβ and Jβ gene usage between two groups, significant differences were observed in almost half of the Vβ and Jβ genes (27/65 Vβ gene and 8/13 Jβ genes, Figures 3A,B), which indicated that the usage patterns of Vβ and Jβ genes were skewed in MN patients. These changes may reflect an altered immune status associated with MN. We also compared the Vβ and Jβ genes between the patients with positive anti-PLA2R antibodies and those with negative antibodies and found that six Vβ genes (Vβ4.2, Vβ6.4, Vβ7.3, Vβ7.5, Vβ13, and Vβ24.1) and three Jβ genes (Jβ1.6, Jβ2.3, and Jβ2.7) had significant differences (P < 0.05). Moreover, we found that Vβ10.2 decreased significantly after therapy in the patients with anti-PLA2R antibodies turning negative. Given that TCRs with similar CDR3 sequences might recognize the same antigenic peptides, we identified CDR3 motifs based on similarities in their CDR3 aa sequences using the CD-HIT program (90% quantile). Two CDR3 motifs were associated with MN with the most significance (Chi-square test, *P* < 0.0001, Figure 4), which might be valuable in immunotherapy.

### The Usage Patterns of Vβ Genes Are Associated With Clinical Response

**Figure 5:**
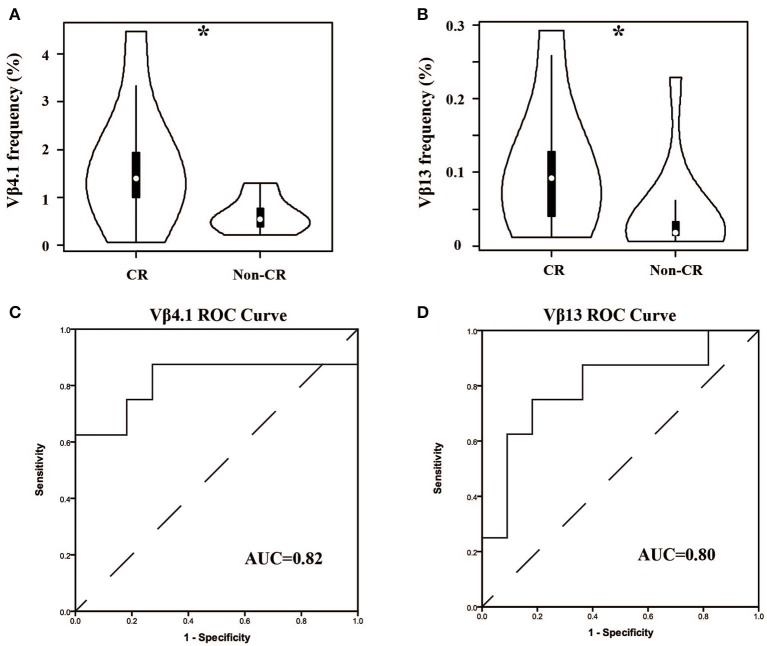
Comparison of Vβ4.1 and Vβ13 gene segments used in patients with or without urinary protein after 6-months therapy. **(A,B)** The usage frequencies of Vβ4.1 and Vβ13 gene segments in CR group and non-CR group. **(C,D)** ROC analysis for the frequencies of Vβ4.1 and Vβ13 gene segments in MN patients to separate the two groups. *P < 0.05.

In our cohort, the patients were divided into two groups—complete remission (CR) and non-CR—according to KDIGO after 6 months of treatment. We compared the Vβ and Jβ gene usage in pre-therapy blood between the two groups, and we found that the frequency of Vβ4.1 and Vβ13 were significant higher in the CR group who had good clinical response, and these two Vβ genes had the potential to predict the therapeutic effect (Figures 5A,B). The areas under the curve (AUC) of Vβ4.1 and Vβ13 were 0.82 and 0.80, respectively (Figures 5C,D). Moreover, we also found that the frequency of Vβ4.1 and Vβ13 were significant higher in healthy control than MN patients (Figure 3A), which indicated their potential value in diagnosis and prognosis.

### Dramatic Changes in TCR Repertoire After Treatment Are Associated With Good Outcomes

**Figure 6:**
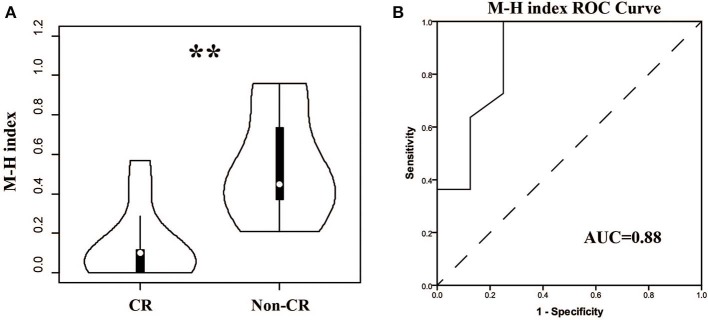
Comparison of the similarities between pre- and post-therapy in CR and non-CR patients after 6-months therapy. **(A)** The Morisita-Horn similarity (MH) index in CR group and non-CR group. **(B)** ROC analysis to separate the two groups. **P < 0.01.

For patients, the TCR repertoire would change after treatment. We calculated the Morisita-Horn similarity index between pre-therapy and post-therapy TCRβ repertoires for every patient. Interestingly, the indexes in the CR group were significant lower (Figure 6, *P* < 0.01, AUC = 0.88). This result indicated that dramatic changes in the TCR repertoire after treatment were associated with good outcomes, which might be due to remission and a change of autoantigens.

## Discussion

In this study, we performed TCRβ repertoire HTS of the PBMC of MN patients in pre- and post- therapy stages, as well as of PBMC of healthy controls, to investigate the relevance of T-cell immunity and the clinical characteristics of MN. Many studies indicated aberration in the usage of the TCRβ V gene and J gene in autoimmune diseases and cancers (15–20). In this study, we found a similar situation in MN patients. The usage pattern of Vβ and Jβ genes was skewed significantly in MN patients, and the diversity of the VJ cassette combination was narrowed, which reflected an altered immune status associated with MN.

As we know, the usage of prednisone and tacrolimus in MN patients has many bad side effects, such as infection, centripetal obesity, osteoporosis, and so on. The prediction of treatment effects before and during therapy could thus prevent the overuse of drugs and minimize the harm to patients. In fact, some studies have reported several predictors of responses to drugs in MN, such as baseline proteinuria, the reduction of proteinuria at month 3, a lower percentage of Tregs at baseline, and an increased percentage of Tregs at day 8 (12, 33). In this study, we found that the TCRβ repertoire could predict the response to treatments, which had not been reported in MN. Two V genes were found to be the candidate biomarkers—Vβ4.1 and Vβ13. High-usage frequencies of them were associated with good outcomes, and their usage frequencies were significant higher in the healthy controls. These results suggested that the T cells in these two TCR subfamilies might be the antigen-specific Tregs that help to counter T-cell-mediated immune responses. Alternatively, they also might mark the effector T cells with altered recognition of an undefined autoantigen, which might skew or attenuate the immune response. There is no evidence for these hypotheses, and further studies are thus needed. We also compared the other clinical characteristics (24 hUP, Serum albumin, APLA2R, eGFR, Serum Creatinine, and Serum Urea Nitrogen) between CR and non-CR patients, and there were no significant differences. It indicated that, in our study, these characteristics could reflect the status of MN, but their prediction potential might be weaker than the TCRβ repertoire. Moreover, limited by the number of samples in our study, validation of the performance of those biomarkers and demonstrating the accuracy and reproducibility of this method by more studies from other laboratories is needed.

In this study, two patients were treated with tacrolimus because they had the contraindication to prednisone, and they did not go into complete remission. If we excluded these two patients, the Vβ4.1 and Vβ13 also had the potential to predict the therapeutic effect (AUC = 0.81 and 0.76). It is very interesting to determine whether patients receiving tacrolimus have similar TCRVβ usage compared to CR and non-CR in patients receiving prednisone. But the number of the patients receiving tacrolimus is much lower than prednisone in the first 6 months. We will extend the follow-up period in our further study to further explore the answer of this question.

In theory, MN is an immune-related disease. If the treatments were effective, the immune status of the body will be different form the pre-treatment, and the TCRβ repertoire will also change significantly; this has been reported in various types of immune-mediated diseases (19, 34, 35). We therefore calculated the similarity of the TCRβ repertoire between pre- and post-therapy in each patient. As expected, the similarity was significantly lower in patients with good outcomes, which indicated that the autoimmune antigen had been inhibited or cleared. However, peripheral blood samples were only collected at 6 months after treatment, and it was impossible to judge whether the change of TCRβ repertoire could reflect the treatment effects earlier than the current biomarkers in use. We plan to focus on it in our future studies.

We separated responders from non-responders in the analyses shown by Figures 1C,D and found no significant correlation between the Shannon entropy index and therapeutic outcome. The Shannon entropy index and the Morisita-Horn similarity index reflect different dimensionalities of the TCR repertoire. The Morisita-Horn similarity index reflects the overlap between two TCR repertoire, and the Shannon entropy index reflects the TCR diversity. In our results, the TCR diversity did not change significantly after therapy, but the overlap between pre- and post-therapy was low, which indicates that, in the responders group, the old T cell clonotypes in the pre-therapy samples disappeared or were exhausted, and many new clonotypes emerged in the post-therapy samples.

A combination of steroids and cytotoxic agents are used as first-line therapy according to KDIGO. But some studies in patients from East Asia showed that the steroid alone was also useful. One study from Japan reported that the steroid alone seems to be useful in Japanese patients, and a remission from heavy proteinuria likely resulted in a favorable outcome (*N* = 949) (36). Another study found that there was a similar treatment response to the steroid alone or in a combination of steroids and cytotoxic agents in Chinese patients, which was contrary to reports in Caucasians (37). Moreover, a study in Korean patients showed that 5-years CR rates of C (corticosteroids alone) and C+C (corticosteroids plus cyclosporine) were 88.5 and 86.2%, respectively. Ten-year event-free rates in these groups were 91.7 and 79.9%, respectively, and they concluded that stepwise treatment using corticosteroids alone and in combination with cyclosporine is warranted in these patients (38).

In summary, we have presented here a comprehensive landscape of the T-cell repertoire in MN, including the restricted VJ repertoire size and skewed usage patterns of Vβ and Jβ genes. The most important discovery was that the TCRβ repertoire similarity between pre- and post-therapy could reflect the clinical outcome, and two Vβ genes in pre-therapy were associated with a therapeutic effect. These findings demonstrated the potential of the TCRβ repertoire as alternative non-invasive biomarkers for the prognosis prediction of MN. However, the independent validation cohort study was still needed.

## Data Availability Statement

The data of this article will be available upon request to the corresponding author.

## Ethics Statement

The studies involving human participants were reviewed and approved by the ethics committee of the Affiliated Foshan Hospital of Sun Yat-Sen University. Written informed consent to participate in this study was provided by the participants' legal guardian/next of kin.

## Author Contributions

YZ, HL, CX, XL, PY, and YK provided patients samples and clinical information. YJ, ZG, ZS, XC, YP, and LZ performed the experiments. YZ and YJ performed the statistical analysis. YZ, YJ, and WL analyzed and interpreted the data. YK and WL designed and supervised the study. YJ and YZ wrote the manuscript. WL carried out an extensive revision. All authors participated in the writing and approval of the manuscript.

### Conflict of Interest

The authors declare that the research was conducted in the absence of any commercial or financial relationships that could be construed as a potential conflict of interest.

## Supplementary Material

Figure S1 Comparison of the age **(A)** and gender **(B)** between healthy controls and MN patients.

Figure S2 No significant correlation between the diversity and clinical characteristics. ns, no significant.

Table S1 Clinical and sequencing information of the patients.

Table S2 The V and J genes usage in the blood samples.

## References

[1] Luo W Olaru F Miner JH Beck LH Jr van der Vlag J Thurman JM. Alternative pathway is essential for glomerular complement activation and proteinuria in a mouse model of membranous nephropathy. Front Immunol. (2018) 9:1433. 10.3389/fimmu.2018.01433 29988342 PMC6023961

[2] Beck LH Jr Salant DJ. Membranous nephropathy: from models to man. J Clin Invest. (2014) 124:2307–14. 10.1172/JCI72270 24892704 PMC4089468

[3] Cattran DC Brenchley PE. Membranous nephropathy: integrating basic science into improved clinical management. Kidney Int. (2017) 91:566–74. 10.1016/j.kint.2016.09.048 28065518

[4] Xu X Wang G Chen N Lu T Nie S Xu G. Long-term exposure to air pollution and increased risk of membranous nephropathy in China. J Am Soc Nephrol. (2016) 27:3739–46. 10.1681/ASN.2016010093 27365535 PMC5118492

[5] Beck LH Jr Bonegio RG Lambeau G Beck DM Powell DW Cummins TD. M-type phospholipase A2 receptor as target antigen in idiopathic membranous nephropathy. N Engl J Med. (2009) 361:11–21. 10.1056/NEJMoa0810457 19571279 PMC2762083

[6] Tomas NM Beck LH Jr Meyer-Schwesinger C Seitz-Polski B Ma H Zahner G. Thrombospondin type-1 domain-containing 7A in idiopathic membranous nephropathy. N Engl J Med. (2014) 371:2277–87. 10.1056/NEJMoa1409354 25394321 PMC4278759

[7] Canadas-Garre M Anderson K McGoldrick J Maxwell AP McKnight AJ. Genomic approaches in the search for molecular biomarkers in chronic kidney disease. J Transl Med. (2018) 16:292. 10.1186/s12967-018-1664-7 30359254 PMC6203198

[8] Hu SL Wang D Gou WJ Lei QF Ma TA Cheng JZ. Diagnostic value of phospholipase A2 receptor in idiopathic membranous nephropathy: a systematic review and meta-analysis. J Nephrol. (2014) 27:111–6. 10.1007/s40620-014-0042-7 24500886

[9] Wang B Zuo K Wu Y Huang Q Qin WS Zeng CH. Correlation between B lymphocyte abnormality and disease activity in patients with idiopathic membranous nephropathy. J Int Med Res. (2011)39:86–95. 10.1177/147323001103900111 21672311

[10] Ozaki T Tomino Y Nakayama S Koide H. Two-color analysis of lymphocyte subpopulations in patients with nephrotic syndrome due to membranous nephropathy. Clin Nephrol. (1992) 38:75–80. 1516283

[11] Zucchelli P Ponticelli C Cagnoli L Aroldi A Beltrandi E. Prognostic value of T lymphocyte subset ratio in idiopathic membranous nephropathy. Am J Nephrol. (1988) 8:15–20. 10.1159/000167547 2967032

[12] Rosenzwajg M Languille E Debiec H Hygino J Dahan K Simon T. B- and T-cell subpopulations in patients with severe idiopathic membranous nephropathy may predict an early response to rituximab. Kidney Int. (2017) 92:227–37. 10.1016/j.kint.2017.01.012 28318628

[13] Hou D Chen C Seely EJ Chen S Song Y. High-throughput sequencing-based immune repertoire study during infectious disease. Front Immunol. (2016) 7:336. 10.3389/fimmu.2016.00336 27630639 PMC5005336

[14] Emerson RO DeWitt WS Vignali M Gravley J Hu J K Osborne E J. Immunosequencing identifies signatures of cytomegalovirus exposure history and HLA-mediated effects on the T cell repertoire. Nat Genet. (2017) 49:659–65. 10.1038/ng.3822 28369038

[15] Tipton CM Fucile CF Darce J Chida A Ichikawa T Gregoretti I. Diversity, cellular origin and autoreactivity of antibody-secreting cell population expansions in acute systemic lupus erythematosus. Nat Immunol. (2015) 16:755–65. 10.1038/ni.3175 26006014 PMC4512288

[16] Gomez-Tourino I Kamra Y Baptista R Lorenc A Peakman M. T cell receptor beta-chains display abnormal shortening and repertoire sharing in type 1 diabetes. Nat Commun:(2017) 8:1792. 10.1038/s41467-017-01925-2 29176645 PMC5702608

[17] Cui JH Jin YB Lin KR Xiao P Chen XP Pan YM. Characterization of peripheral blood TCR repertoire in patients with ankylosing spondylitis by high-throughput sequencing. Hum Immunol. (2018) 79:485–90. 10.1016/j.humimm.2018.03.007 29614337

[18] Jin YB Luo W Zhang GY Lin KR Cui JH Chen XP. TCR repertoire profiling of tumors, adjacent normal tissues, and peripheral blood predicts survival in nasopharyngeal carcinoma. Cancer Immunol Immunother. (2018) 67:1719–30. 10.1007/s00262-018-2237-6 30155576 PMC11028245

[19] Lin KR Pang DM Jin YB Hu Q Pan YM Cui JH. Circulating CD8(+) T-cell repertoires reveal the biological characteristics of tumors and clinical responses to chemotherapy in breast cancer patients. Cancer Immunol Immunother. (2018) 67:1743–52. 10.1007/s00262-018-2213-1 30167861 PMC11028329

[20] Cui JH Lin KR Yuan SH Jin YB Chen XP Su XK. TCR Repertoire as a novel indicator for immune monitoring and prognosis assessment of patients with cervical cancer. Front Immunol. (2018) 9:2729. 10.3389/fimmu.2018.02729 30524447 PMC6262070

[21] Gordon CE Balk EM Francis JM. Summary of the 2018 kidney disease improving global outcomes (KDIGO) guideline on hepatitis C in chronic kidney disease. Semin Dial. (2019) 32:187–95. 10.1111/sdi.12768 30496617

[22] Eckardt KU Kasiske BL. Kidney disease: improving global outcomes. Nat Rev Nephrol. (2009) 5:650–7. 10.1038/nrneph.2009.153 19786993

[23] Ruggiero E Nicolay JP Fronza R Arens A Paruzynski A Nowrouzi A. High-resolution analysis of the human T-cell receptor repertoire. Nat Commun. (2015) 6:8081. 10.1038/ncomms9081 26324409 PMC4569693

[24] Giudicelli V Chaume D Lefranc MP. IMGT/GENE-DB: a comprehensive database for human and mouse immunoglobulin and T cell receptor genes. Nucleic Acids Res. (2005) 33:D256–61. 10.1093/nar/gki010 15608191 PMC539964

[25] Shannon CE. The mathematical theory of communication. 1963. MD Comput. (1997) 14:306–17. 9230594

[26] Schneider-Hohendorf T Mohan H Bien CG Breuer J Becker A Gorlich D. CD8(+) T-cell pathogenicity in Rasmussen encephalitis elucidated by large-scale T-cell receptor sequencing. Nat Commun. (2016) 7:11153. 10.1038/ncomms11153 27040081 PMC4822013

[27] Sims JS Grinshpun B Feng Y Ung TH Neira JA Samanamud JL. Diversity and divergence of the glioma-infiltrating T-cell receptor repertoire. Proc Natl Acad Sci USA. (2016) 113:E3529–37. 10.1073/pnas.1601012113 27261081 PMC4922177

[28] Hindley JP Ferreira C Jones E Lauder SN Ladell K Wynn KK. Analysis of the T-cell receptor repertoires of tumor-infiltrating conventional and regulatory T cells reveals no evidence for conversion in carcinogen-induced tumors. Cancer Res. (2011) 71:736–46. 10.1158/0008-5472.CAN-10-1797 21156649 PMC3128990

[29] Su S Liao J Liu J Huang D He C Chen F. Blocking the recruitment of naive CD4(+) T cells reverses immunosuppression in breast cancer. Cell Res. (2017) 27:461–82. 10.1038/cr.2017.34 28290464 PMC5385617

[30] Freeman JD Warren RL Webb JR Nelson BH Holt RA. Profiling the T-cell receptor beta-chain repertoire by massively parallel sequencing. Genome Res. (2009) 19:1817–24. 10.1101/gr.092924.109 19541912 PMC2765271

[31] Chen Y Xu Y Zhao M Liu Y Gong M Xie C. High-throughput T cell receptor sequencing reveals distinct repertoires between tumor and adjacent non-tumor tissues in HBV-associated HCC. Oncoimmunology. (2016) 5:e1219010. 10.1080/2162402X.2016.1219010 27853640 PMC5087304

[32] Liaskou E Klemsdal Henriksen EK Holm K Kaveh F Hamm D Fear J. High-throughput T-cell receptor sequencing across chronic liver diseases reveals distinct disease-associated repertoires. Hepatology. (2016) 63:1608–19. 10.1002/hep.28116 26257205

[33] Li S Wang L Zhang M Zhou W Fang W Wang Q. Clinical predictors of response to prednisone plus cyclophosphamide in patients with idiopathic membranous nephropathy. Nephron. (2017) 135:87–96. 10.1159/000448291 27974710

[34] Liu YY Yang QF Yang JS Cao RB Liang JY Liu YT. Characteristics and prognostic significance of profiling the peripheral blood T-cell receptor repertoire in patients with advanced lung cancer. Int J Cancer. (2019) 145:1423–31. 10.1002/ijc.32145 30664810

[35] Cha E Klinger M Hou Y Cummings C Ribas A Faham M. Improved survival with T cell clonotype stability after anti-CTLA-4 treatment in cancer patients. Sci Transl Med. (2014) 6:238ra70. 10.1126/scitranslmed.3008211 24871131 PMC4558099

[36] Shiiki H Saito T Nishitani Y Mitarai T Yorioka N Yoshimura A Research group on progressive renal diseases in: prognosis and risk factors for idiopathic membranous nephropathy with nephrotic syndrome in Japan. Kidney Int. (2004) 65:1400–7. 10.1111/j.1523-1755.2004.00518.x 15086481

[37] Tang S Chan TM Cheng IK Lai KN. Clinical features and treatment outcome of idiopathic membranous nephropathy in Chinese patients. QJM. (1999) 92:401–6. 10.1093/qjmed/92.7.401 10627890

[38] Shin DH Lee MJ Oh HJ Koo HM Doh FM Kim HR. Stepwise treatment using corticosteroids alone and in combination with cyclosporine in korean patients with idiopathic membranous nephropathy. Yonsei Med J. (2013) 54:973–82. 10.3349/ymj.2013.54.4.973 23709434 PMC3663215

