# Supplementary figures and images for: The Landscape and Prognosis Potential of the T-Cell Repertoire in Membranous Nephropathy
Source: Front Immunol. 2020 Mar 10;11:387. doi: 10.3389/fimmu.2020.00387 (PMC7076165; doi:10.3389/fimmu.2020.00387)

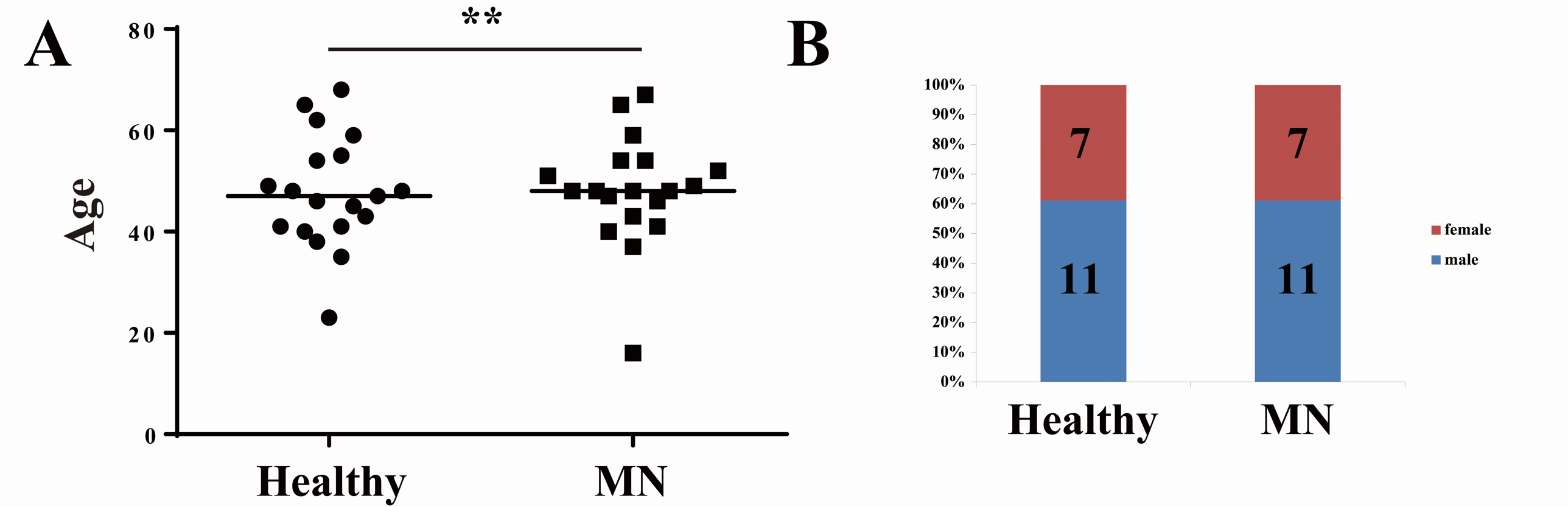

Supplement: Figure S1 — Comparison of the age (A) and gender (B) between healthy controls and MN patients. [file Image_1.TIF]

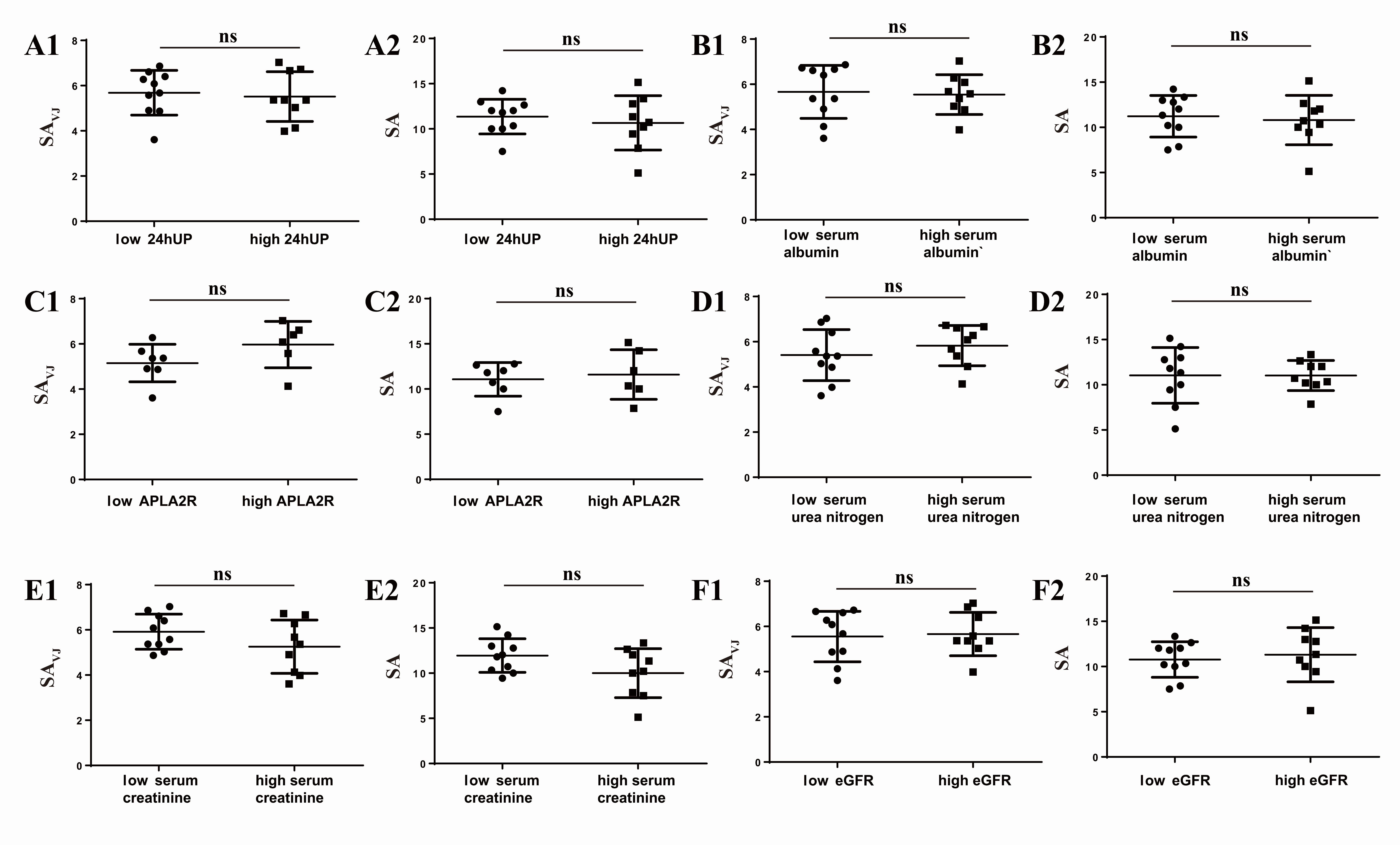

Supplement: Figure S2 — No significant correlation between the diversity and clinical characteristics. ns, no significant. [file Image_2.TIF]
